# Abnormalities in Brain and Muscle Microstructure and Neurochemistry of the DMD Rat Measured by *in vivo* Diffusion Tensor Imaging and High Resolution Localized ^1^H MRS

**DOI:** 10.3389/fnins.2020.00739

**Published:** 2020-07-14

**Authors:** Su Xu, Shiyu Tang, Xin Li, Shama R. Iyer, Richard M. Lovering

**Affiliations:** ^1^Department of Diagnostic Radiology and Nuclear Medicine, University of Maryland School of Medicine, Baltimore, MD, United States; ^2^Center for Advanced Imaging Research, University of Maryland School of Medicine, Baltimore, MD, United States; ^3^Department of Orthopaedics, University of Maryland School of Medicine, Baltimore, MD, United States

**Keywords:** duchenne muscular dystrophy, rat model, neuroimaging, magnetic resonance imaging, ^1^H magnetic resonance spectroscopy, diffusion tensor imaging

## Abstract

Duchenne muscular dystrophy (DMD) is an X-linked disorder caused by the lack of dystrophin with progressive degeneration of skeletal muscles. Most studies regarding DMD understandably focus on muscle, but dystrophin is also expressed in the central nervous system, potentially resulting in cognitive and behavioral changes. Animal models are being used for developing more comprehensive neuromonitoring protocols and clinical image acquisition procedures. The recently developed DMD rat is an animal model that parallels the progressive muscle wasting seen in DMD. Here, we studied the brain and temporalis muscle structure and neurochemistry of wild type (WT) and dystrophic (DMD) rats using magnetic resonance imaging and spectroscopy. Both structural and neurochemistry alterations were observed in the DMD rat brain and the temporalis muscle. There was a decrease in absolute brain volume (*WT* = 1579 mm^3^; *DMD* = 1501 mm^3^; *p* = 0.039, Cohen’s *d* = 1.867), but not normalized (*WT* = 4.27; *DMD* = 4.02; *p* = 0.306) brain volume. Diffusion tensor imaging (DTI) revealed structural alterations in the DMD temporalis muscle, with increased mean diffusivity (MD), axial diffusivity (AD), and radial diffusivity (RD). In the DMD rat thalamus, DTI revealed an increase in fractional anisotropy (FA) and a decrease in RD. Smaller normalized brain volume correlated to severity of muscle dystrophy (*r* = −0.975). Neurochemical changes in the DMD rat brain included increased GABA and NAA in the prefrontal cortex, and GABA in the hippocampus. Such findings could indicate disturbed motor and sensory signaling, resulting in a dysfunctional GABAergic neurotransmission, and an unstable osmoregulation in the dystrophin-null brain.

## Introduction

Duchenne muscular dystrophy (DMD) is an X-linked muscle disorder that presents clinically with significant and progressive muscle wasting and loss of muscular function (Brooke et al., 1989; McDonald et al., 1995; Lovering et al., 2005), due to the absence of the protein, dystrophin. Dystrophin is a large sarcolemma-associated protein expressed in striated muscle (Tinsley et al., 1994; Blake et al., 1996). Our understanding about the function of dystrophin has been derived from studies of dystrophin-deficient animals, with the most common model being the *mdx* mouse.

Dystrophin is also expressed in the central nervous system (CNS), but there is a dearth of knowledge regarding its role in the brain. Cognitive impairment, including mental retardation (Cotton et al., 2005) can occur in DMD patients (Duchenne, 1861; Anderson et al., 2002; Nardes et al., 2012). This can include diminished memory function, especially short term memory (Hinton et al., 2007; Wingeier et al., 2011), as well as language impairments, long-term memory problems, and limited executive functions (Cotton et al., 2001, 2005; Wicksell et al., 2004; Marini et al., 2007). Both CT and *in vivo* ^1^H MRS studies indicate a slow, progressive cerebral degeneration in DMD patients (Yoshioka et al., 1980; Uchino et al., 1994; Rae et al., 1998; Chen et al., 1999), with findings of cortical atrophy and ventricular dilation in older patients, and patients with large amounts of physical disability (Yoshioka et al., 1980).

In muscle, dystrophin protects the sarcolemma against mechanical stress (Koenig et al., 1988) and contributes to force and signal transduction (Ervasti and Campbell, 1993; Petrof et al., 1993; Lovering and De Deyne, 2004). Dystrophin also regulates intracellular calcium and the cascade of calcium-related events (Lansman and Franco, 1991; Lovering et al., 2009), the aggregation of neurotransmitter receptors (Kong and Anderson, 1999; Pratt et al., 2013), and reactive oxygen species (Whitehead et al., 2006; Khairallah et al., 2012). In the brain, various dystrophin isoforms are distributed unequally, but dystrophin is expressed in the post-synaptic neurons of the cortex, hippocampus, and cerebellum, which are regions integral to cognition (Cyrulnik and Hinton, 2008). Thus, cognitive impairment in DMD patients could be due to its absence. However, the specific function of dystrophin in brain is not fully understood. In muscle, dystrophin has functional and structural roles. Identifying how dystrophin’s absence affects the brain would be helpful in monitoring disease progression or effectiveness of therapies that are being developed for DMD.

The most commonly studied animal model of DMD is the *mdx* mouse, which similar to patients with DMD, lacks dystrophin (Sicinski et al., 1989). However, even though *mdx* mice have muscle pathology, the phenotype is much less severe than patients with DMD. Thus, the utility of the *mdx* mouse as a model for DMD is questionable (Allamand and Campbell, 2000). Novel rat models of DMD have become available (Larcher et al., 2014; Nakamura et al., 2014), but have still not been fully characterized. The rat model appears to better mimic DMD than the *mdx* mouse, with progressive muscle fibrosis, fatty infiltration, muscle weakness, decreased activity, and altered diastolic function (Larcher et al., 2014; Nakamura et al., 2014). A recent report showed that the DMD rat shows significant changes in neuromotor behavior (Caudal et al., 2020). The DMD rat more closely parallels the phenotype of DMD than *mdx* mice in terms of histology and life span, but there are no imaging studies of the rat DMD brain. In this brief research report, we used *in vivo* magnetic resonance imaging (MRI), diffusion tensor imaging (DTI) and high resolution localized ^1^H magnetic resonance spectroscopy (^1^H MRS) to investigate alterations in brain structure and neurochemicals in the DMD rat brain and adjacent temporalis muscle. The use of these *in-vivo* neuroimaging technologies will be helpful in clarifying the function of dystrophin in the brain. Furthermore, the development of these neuroimaging techniques is important as non-invasive tools for diagnosis, monitoring disease progression, planning rehabilitation, and determining the effectiveness of therapeutic interventions.

## Methods

### Animals

DMD rats were generated using a CRISPR-based approach targeting exon 22 to exon 26 of dystrophin (gRNA pairs GTCTAA TAGTAGGTGATAAGAGG and CAGCTCTTGTACCCGATTG CTGG), resulting in a ∼1080 bp mutant dystrophin mRNA that is undetectable. Two-month old dystrophic (DMD, *N* = 4, 356 ± 26 g) and age-matched littermate wild type (WT, *N* = 4, 384 ± 29 g) male rats were used. Body weight was not significantly different between DMD and WT rats. All experimental procedures were approved by the University of Maryland School of Medicine Institutional Animal Care and Use Committee.

### MRI Protocol

The MRI/MRS experiments were performed on a Bruker BioSpec 70/30USR Avance III 7T scanner. A Bruker four-element ^1^H surface coil array was used as the receiver and a Bruker 72 mm linear-volume coil as the transmitter. Each rat was anesthetized in an animal chamber using a gas mixture of O_2_ (1 L/min) and isoflurane (3%) then later maintained at 1–2% isoflurane during scanning. An MR compatible small-animal monitoring system was used to monitor the animal respiration rate and body temperature. The animal body temperature was maintained at 35–37°C using warm water circulation.

**FIGURE 1:**
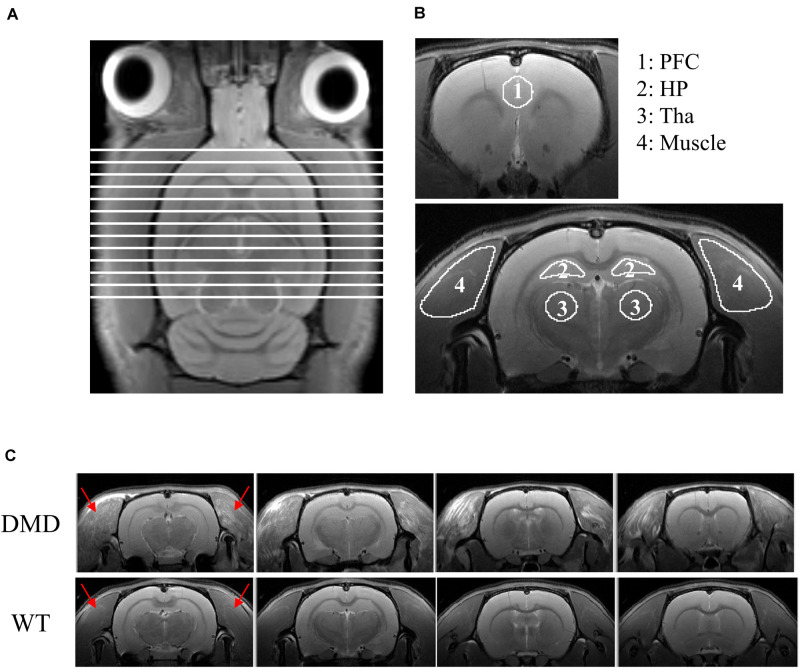
**(A)** T_2_-weighted image of a rat brain showing all slices included for the calculation of brain volume. **(B)** Regions of interest (ROIs) used for DTI data analysis. ROIs were drawn in the prefrontal cortex (PFC; ROI 1), hippocampus (HP; ROI 2), thalamus (Tha, ROI 3) and temporalis muscle (ROI 4). **(C)** RARE T_2_-weighted MRI axial view of the muscle surrounding the cranium (temporalis muscle, red arrows) of the head of each wild-type (WT) and dystrophic (DMD) rat. In the WT rats, the temporalis muscles were homogeneously dark with no focal hyperintense regions, whereas in the DMD rats, the temporalis muscles were heterogeneous, with multiple, unevenly distributed focal hyperintensities. There were no apparent anatomic differences between WT and DMD brains. RARE: rapid acquisition with relaxation enhancement sequence.

T_2_-weighted anatomic head (including brain and the surrounding muscle tissues) images were obtained using a 2D rapid acquisition with relaxation enhancement (RARE) sequence in the axial plane [repetition time (TR)/echo time (TE) = 4600/30 ms, RARE factor = 4, field of view (FOV) = 32 × 32 mm^2^, slice thickness = 1 mm, image matrix = 320 × 320, in-plane resolution = 100 × 100 μm^2^, number of averages (NA) = 2, number of slices = 16]. Brain volume analysis was performed in MIPAV (Medical Image Processing, Analysis, and Visualization^1^). Skull-stripping was performed manually on images. The total brain volume was calculated by adding the slice-by-slice volume from 13 slices that were consistently acquired across animals as shown in Figure 1A.

*In vivo* DTI images on rat heads were acquired with a gradient echo-planar imaging (EPI) sequence (TR/TE = 2,500/18.82 ms, diffusion directions = 30, NA = 1, image matrix = 92 × 92, in-plane resolution = 348 × 348 μm^2^, the FOV was the same as T_2_-weighted images). Two *b*-values (1,000 and 2,000 s/mm^2^) were acquired for each direction. Five additional images at *b* = 0 s/mm^2^ were also acquired. Typically for routine brain evaluations, the *b*-values (a measure of the sensitivity to diffusion) in DTI experiments are at 1,000 s/mm^2^ (Xu et al., 2011; Zhuo et al., 2012). The *b* = 2000 s/mm^2^ was added in the current study to test whether better characterized gray matter microstructure would yield differences in brain maturation (Cheung et al., 2009; Zhuo et al., 2012). However, no significant differences were found in the current age group of DMD and WT rats. The acquisition of multiple images acquired at *b* = 0 s/mm^2^ was utilized to obtain a strong signal to noise ratio for non-diffusion weighted map to improve the estimation of diffusion parameters (Jones et al., 1999).

Custom-made diffusion kurtosis software (Xu et al., 2013) was used to generate the maps of mean diffusivity (MD), axial diffusivity (AD), radial diffusivity (RD), and fractional anisotropy (FA). MD measures the average water diffusion within the head. AD measures the water diffusion along the neuronal axons. RD measures the water diffusion perpendicular to the axons. FA measures the degree of diffusion anisotropy of the head. The regions of interest (ROIs), including prefrontal cortex, hippocampus, thalamus, and the temporalis muscle were manually defined on the FA images while using the T_2_-weighted image for anatomic reference in FSLeyes^2^ (Figure 1B). Values of MD, AD, RD, and FA were extracted, respectively, from each generated map using the manually defined ROIs. The details of this procedure have been published previously (Zhuo et al., 2012).

A ^1^H short-TE Point-RESolved Spectroscopy (PRESS) pulse sequence (Xu et al., 2013) (TR/TE = 2,500/10 ms, NA = 360) was used for MRS data acquisition with the voxel centered on the prefrontal cortex (PFC, 3 mm × 3 mm × 3 mm) and hippocampus (HP, 8 mm × 2 mm × 2 mm), respectively. The unsuppressed water signal from each of the prescribed voxels was obtained to serve as a reference for determining the specific metabolite concentrations. Quantification of the MRS was based on frequency domain analysis using a “Linear Combination of Model spectra” (LCModel) (Provencher, 1993). Absolute concentrations were estimated with the LCModel automatic procedure (version 6.3-0G).

### Statistics

All *in vivo* MRI and MRS measurements between DMD and WT rats were compared using *t*-tests with an alpha level set at *p* < 0.05. Cohen’s d was used to measure effect size. Correlations between normalized brain volume, MRS measurements, and DTI parameters of MD, AD, RD, and FA were calculated using Pearson’s correlation coefficient.

## Results

**FIGURE 2:**
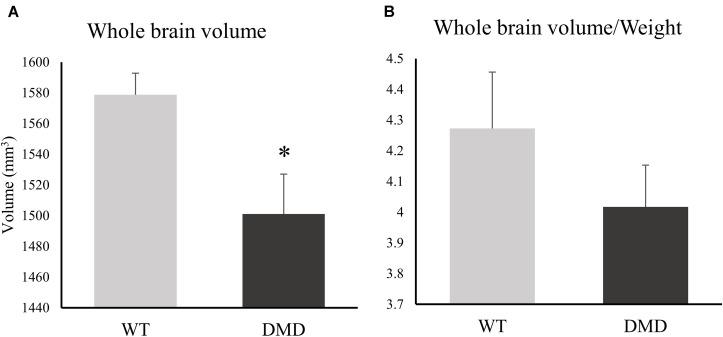
**(A)** Bar graph showing differences between the whole brain volume of dystrophic (DMD) and wild-type (WT) rats. **(B)** Bar graph showing differences between the normalized brain volume of DMD and WT animals. *significantly different from the corresponding values in WT rats (un-paired *t*-test, *p* < 0.05).

DMD rats clearly had muscle lesions (Figure 1C), which showed a similar pattern to what we have reported in *mdx* mice (Xu et al., 2015). The MRI of WT temporalis muscles were homogeneous, but these muscles of DMD rats showed signal heterogeneity, with numerous focal hyperintensities clearly present. There were no apparent anatomical differences in T_2_-weighted images of the brains between the WT and DMD rats (Figure 1C). A significant reduction of whole brain volume was detected in DMD rats compared to WT rats (*p* = 0.039, Cohen’s *d* = 1.867) (Figure 2A), however, this difference was not significant when the brain volume was normalized to body weight (*p* = 0.306) (Figure 2B).

**FIGURE 3:**
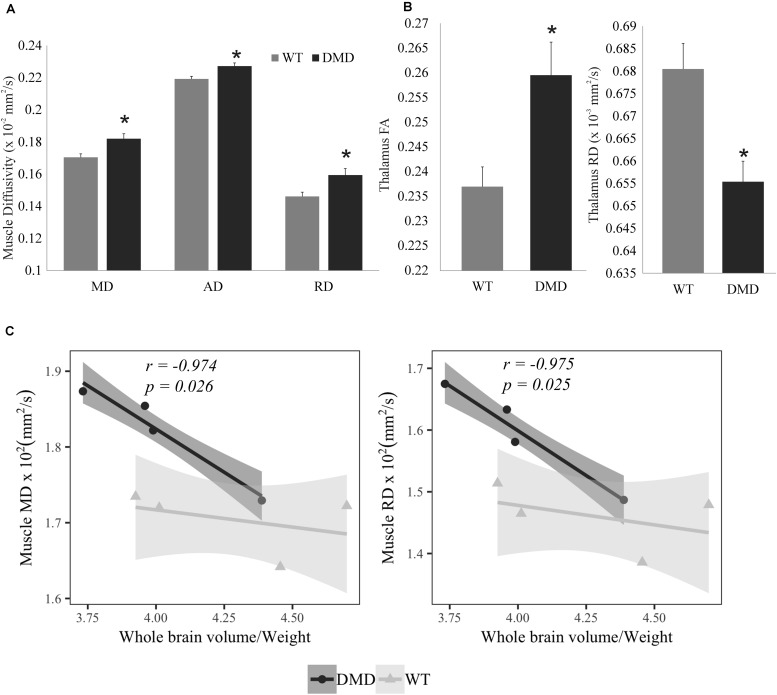
**(A)** Bar graph showing the increased values in muscle mean diffusivity (MD), axial diffusivity (AD), and radial diffusivity (RD) in dystrophic (DMD) rats compared to wild-type (WT) rats. **(B)** Bar graph showing that fractional anisotropy (FA) was significantly increased, while RD was reduced in the thalamus of DMD rats compared to WT rats. **(C)** Correlation plots between normalized whole brain volume and both muscle MD (left) and muscle RD (right) in DMD and WT rats. *Significantly different from the corresponding value in WT rats (un-paired *t*-test, *p* < 0.05).

All of the diffusivities were significantly increased in the temporalis muscles of the DMD rats compared to WT rats (MD: *p* = 0.024, Cohen’s *d* = 2.255; AD: *p* = 0.024, Cohen’s *d* = 2.234; RD: *p* = 0.034, Cohen’s *d* = 1.199) (Figure 3A). FA significantly increased (*p* = 0.028, Cohen’s *d* = 2.041) while RD significantly decreased (*p* = 0.014, Cohen’s *d* = 2.5) in the thalamus of DMD rats compared to WT rats (Figure 3B), which could signify either increased myelination, or decreased fiber diameters. A correlation analysis was performed between the normalized brain volume and the MD, AD, and RD value of temporalis muscles in WT and DMD rats using Pearson’s correlation analysis. Significant negative correlation was detected in DMD rats between normalized brain volume and muscle MD (*r* = −0.974, *p* = 0.026), and normalized brain volume and muscle RD (*r* = −0.975, *p* = 0.025), respectively (Figure 3C). No significant correlations were observed in WT rats. This finding suggests that the level of brain atrophy is associated with the severity of dystrophic muscle in DMD rats. No significant difference between WT and DMD rats was detected in any of the diffusion measures in prefrontal cortex and hippocampus (data not shown).

**FIGURE 4:**
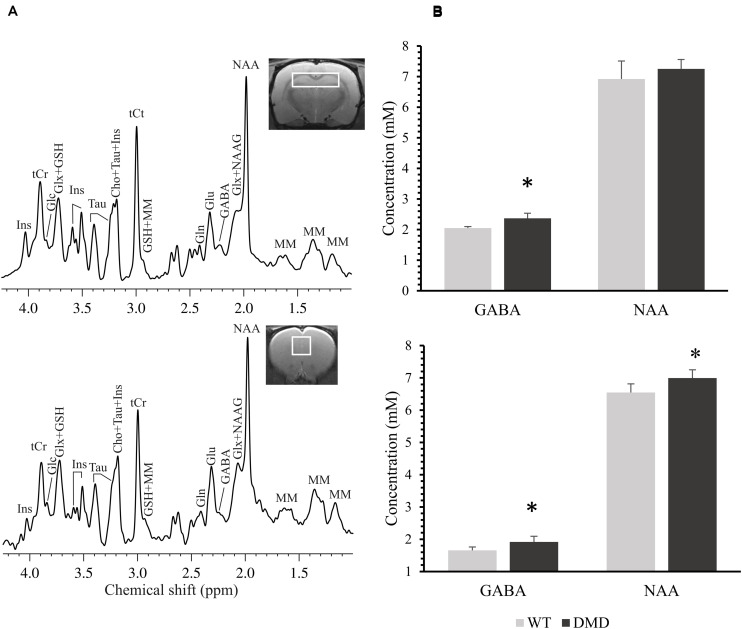
**(A)** Representative *in vivo* ^1^H MR spectra acquired from the prefrontal cortex (PFC, bottom) and the hippocampus (HP, top) of a dystrophic (DMD) rat brain. Main metabolites shown in the spectra are: γ-aminobutyric acid (GABA), glutamine (Gln), glutamate (Glu), glucose (Glc), glutathione (GSH), *myo*-inositol (Ins), *N*-acetylaspartate (NAA), *N*-acetylaspartateglutamate (NAAG), taurine (Tau), total creatine (tCr; phosphocreatine + creatine), choline (Cho), glutamate/glutamine complex (Glx), and macro molecules (MM). **(B)** Comparison of the neurochemical concentrations (mM) between wild-type (WT) and DMD rats in PFC (bottom) and HP (top). Data are expressed as mean ± standard error. *Significantly different from the corresponding value in WT rats (un-paired *t*-test, *p* < 0.05).

Representative high resolution localized *in vivo* MRS spectra from the PFC and HP of a DMD rat are shown in Figure 4A. Several neurochemicals were detected, such as total creatine (tCr, creatine + phosphocreatine), γ-aminobutyric acid (GABA), glucose (Glc), glutamate (Glu), glutamine (Gln), glutathione (GSH), *myo-*inositol (Ins), *N*-acetyl-aspartate (NAA), *N*-acetylaspartateglutamate (NAAG), taurine (Tau), Choline (Cho), and macro molecules (MM). Compared to WT mice, DMD rats demonstrated significant elevations in GABA (*p* = 0.045) and NAA (*p* = 0.044) in the PFC, as well as a marginal elevation in GABA (*p* = 0.062) in the HP (Figures 4A,B). No significant differences were seen in other neurochemicals from these regions. The relationship between MRS and DTI results was also performed, but no significant correlation was found (data not shown).

## Discussion

This brief study demonstrated that multi-modal MR neuroimaging modalities can identify changes in the structure (MRI/DTI) and neurobiochemistry (MRS) of muscle and brain in young DMD rats. DMD rats had smaller brain volumes and body weights, although there was no significant difference in normalized brain volume. Interestingly, a recent study looking at aged *mdx* mice showed a progressive, age-dependent decline in cognitive function (Bagdatlioglu et al., 2020). The authors suggested that the absence of dystrophin causes a late onset neurodegeneration that is not readily detected in the *mdx* mice because most testing has been performed in relatively young animals before the onset of this degeneration. Such a finding is consistent with MRI findings in older DMD patients, showing reduced total brain volume compared to healthy controls (although such findings are difficult to separate from effects of steroid therapy) (Doorenweerd et al., 2014). Although we did not assess aged rats, the slight reduction in total brain volume in the DMD rat might reflect a phenotype that, compared to *mdx* mice, more closely resembles DMD.

Mastication is impaired in DMD patients, for example patients have a decrease in normal bite force. Thus, it’s plausible that the reduced body weights of the DMD rats compared to controls could be due to reduced food consumption secondary to the muscle pathology in the temporalis muscles (Bagdatlioglu et al., 2020), although this was not assessed.

MD, AD, and RD were significantly increased in the temporalis muscles of the DMD rats compared to WT rats, MD and RD values had significant negative correlations to normalized brain volume in DMD rats, but not in WT rats. This finding suggests that the level of brain atrophy is associated with the severity of muscular dystrophy in DMD rats. The structural alterations seen in DMD rats included increased FA and decreased RD in thalamus, a structure that has extensive nerve connections to the cerebral cortex and the midbrain. The primary function of the thalamus is to relay motor and sensory signals to and from the cerebral cortex. Thus, the microstructural alteration in the thalamus may reflect the movement disorder caused by the muscular dystrophy.

The changes in concentrations of neurochemicals in the DMD rat brains were in the HP and PFC regions, where dystrophin is normally present (Caudal et al., 2020). Elevations in GABA (in PFC and HP) and NAA (in PFC) were observed in DMD rats. A disruption in the amount or relative proportions of these neurochemicals likely has consequences on dystrophin’s functions, such as GABAergic neurotransmission and osmoregulation in the brain.

The elevations in GABA in the both PFC and HP in the DMD rat brains may indicate a dysfunction of GABAergic neurotransmission. It is known that full-length dystrophin co-localizes with GABA_A_ receptors in the mouse cerebellum, cerebral cortex and hippocampus (Knuesel et al., 1999). Dystrophin likely contributes to neurotransmitter receptor aggregation, potentially due to the reduction of GABA_A_ resulting in the elevation of GABA in the brain (Knuesel et al., 1999; Kueh et al., 2011).

NAA is a known osmolyte, providing ∼7% of neuronal osmolarity (Baslow, 2000). Missing cerebral dystrophin may lead to modification of the microenvironment of the neurons and thus may result in a perturbed osmoregulation. The NAA elevation may work as a beneficial compensatory mechanism in the brain.

The subcellular distribution of dystrophin neurons is restricted and varies within the brain, including in the rat (Caudal et al., 2020). If dystrophin can play different roles in different areas of the brain, this could explain why our findings are restricted to some, and not all, brain regions. Interestingly, our findings were restricted to brain regions known to be related to cognitive function. Since distribution of dystrophin is limited in the brain, this could explain why only mild cognitive dysfunction is seen in patients with DMD. Here, we report changes in DTI parameters and GABA and NAA in several brain areas. Such findings indicate small brain volume that correlated to severity of muscle dystrophy, disturbed motor and sensory signal sending, dysfunction of GABAergic neurotransmission, and perturbed osmoregulation in the DMD rat brain. The prefrontal cortex and hippocampus are two regions that show the absence of dystrophin and feasibility of MRS. Therefore, we put these two regions in the localized MRS protocol. Now that we know from the DTI results that the microstructure of the thalamus can be affected by DMD, we will add the thalamus as a target region in our future study for MRS.

Our results in this study on DMD rat brain reflect similar findings found in both the *mdx* mouse model and patients with DMD. Behavioral studies indicate learning impairments in *mdx* mice (Muntoni et al., 1991; Vaillend et al., 1995; Perronnet et al., 2012). A handful of studies that have explored *in vivo* ^1^H MRS indicate low ratios of Cr/Cho and NAA/Cho in *mdx* brains compared to wild type controls (Tracey et al., 1996). Using MRI and MRS, we previously reported structural and biochemical changes in brains of 7-month old *mdx* mice (Xu et al., 2015). Our results showed enlarged lateral ventricles in *mdx* brains when compared to WT. Other structural alterations were observed by *ex vivo* DTI. In the prefrontal cortex, elevations in diffusivities were detected in the prefrontal cortex and a reduction of FA was measured in the hippocampus. Biochemical changes included elevations in phospfholine and glutathione, and a reduction in GABA in the hippocampus of the *mdx* mice. In addition, we found an elevation in taurine in the prefrontal cortex. Such findings indicate a regional structural change, altered cellular antioxidant defenses, modified GABAergic neurotransmission, and disrupted osmoregulation in the brain lacking dystrophin. Significant increases in ratios of choline-containing compounds to *N*-acetylaspartate (Cho/NAA) and Cho/Cr have been detected in the cerebellum of DMD boys compared to age-matched controls (Rae et al., 1998). A progressive nature was further reflected by a larger effect in 12 year old patients with DMD than in 8 year old patients with DMD. Conversely, another study showed a significant decrease in absolute Cho levels in both the cerebellum and the temporo-parietal cortex of the DMD patients (Kreis et al., 2011).

The impact of dystrophin’s absence on behavior has also recently been studied in the DMD rat, showing clear alterations in overall neuromotor function (Caudal et al., 2020). The current study sheds further light on dystrophin’s role in the brain. Replacing dystrophin in the brain would be the most logical solution to resolving cognitive issues, but such a therapy is not imminent, despite ongoing efforts (Goyenvalle et al., 2015). The non-invasive imaging methods employed here could be effective in monitoring the efficiency of the potential therapeutic agents, including the brain if such treatments are developed.

In summary, we studied the structure (brain and muscle) and neuro biochemistry of healthy and dystrophic rats with MRI/MRS and found in dystrophic rats: smaller brain volume that correlated to severity of muscular dystrophy, alterations in diffusion of the muscle and thalamus, and several biochemical alterations in prefrontal cortex and hippocampus.

## Data Availability Statement

All datasets generated for this study are included in the article.

## Ethics Statement

The animal study was reviewed and approved by the University of Maryland School of Medicine Institutional Animal Care and Use Committee.

## Author Contributions

SX and RL contributed to the conception and design. XL and ST contributed to the acquisition and analysis of data. SX, ST, SI, and RL contributed to the interpretation of the results. All authors contributed to the manuscript writing.

## Conflict of Interest

The authors declare that the research was conducted in the absence of any commercial or financial relationships that could be construed as a potential conflict of interest.

## Footnotes

1 https://mipav.cit.nih.gov/

2 https://git.fmrib.ox.ac.uk/fsl/fsleyes/fsleyes
